# Preliminary Clinical Evaluation of Intrafraction Prostate Displacements for Two Immobilization Systems

**DOI:** 10.7759/cureus.10206

**Published:** 2020-09-02

**Authors:** Farshad Mostafaei, Shona T Dougherty, Russell J Hamilton

**Affiliations:** 1 Radiation Oncology, Augusta University Medical College of Georgia, Augusta, USA; 2 Radiation Oncology, University of Arizona, Tucson, USA; 3 Radiation Oncology/Medical Physics, Banner University Medical Center/University of Arizona, Tucson, USA

**Keywords:** prostate, immobilization, hypofractionation, sbrt, intrafraction motion, intrafraction

## Abstract

Immobilization systems and their corresponding set-up errors influence the clinical target volume to the planning target volume (CTV-PTV) margins, which is critical for hypofractionated prostate stereotactic body radiotherapy (SBRT). This preliminary study evaluates intrafraction prostate displacement for two immobilization systems (A and B). Six consecutive patients having localized prostate cancer and implanted prostate marker seeds were studied. Planar X-ray images were acquired pre- and post-treatment to find the intrafraction prostate displacement. The average absolute displacements (lateral, longitudinal, vertical) were 0.9 ± 0.4 mm, 1.7 ± 0.1 mm, 1.3 ± 0.3 mm (system A), and 0.5 ± 0.2 mm, 0.6 ± 0.1 mm, 0.8 ± 0.3 mm (system B), with average three-dimensional displacements of 2.6 ± 0.2 mm (system A) and 1.3 ± 0.2 mm (system B). The computed CTV-PTV margins (lateral, longitudinal, vertical) were 2.5 mm, 2.5 mm, 3.6 mm and 1.4 mm, 1.6 mm, 2.4 mm for systems A and B, respectively. This suggests that the immobilization system influences intrafraction prostate displacement and, therefore, the margins applied. However, the margins found for both systems are comparable to the margins used for hypofractionated prostate SBRT.

## Introduction

Prostate carcinoma is a prevalent malignant disease in men [1]. In general, prostate cancer can be treated by external beam radiotherapy while sparing organs-at-risk (OARs), such as the rectum, small bowel, bladder, bilateral femoral heads, and penile bulb, from high-dose radiation [2-3].

Studies have emphasized the importance of immobilization to reduce set-up uncertainties [4]. By reducing set-up uncertainties, the likelihood of covering the target improves and the likelihood of irradiating normal tissues is reduced [5]. For prostate cancer treatment, improved set-up uncertainties will result in smaller field margins, higher local control rates, and reduce the occurrence of normal tissue toxicities such as diarrhea and cystitis [2,6].

Ideally, patient positioning and immobilization would place a patient in the same position during treatment as during simulation when the data used for treatment planning was collected. This requires that a patient’s position would be reproducible for every treatment fraction (no interfraction variation) and that, once positioned, the patient’s position would not change during treatment (no intrafraction variation). Immobilization systems are used to improve the accuracy of the initial patient placement and to restrict patient motion during treatment, seeking to minimize both interfraction and intrafraction variations, which are inevitable in practice [7-9]. Intensity-modulated radiation therapy (IMRT) (which involves high-dose gradients, often close to critical organs) places increased demands upon precise, reproducible positioning and rigid immobilization. Without careful training in the fabrication of custom treatment devices, significant geometric errors in radiotherapy treatment can occur. Immobilization systems and their corresponding set-up errors influence the CTV to PTV margins and, therefore, may result in undesirable treatment outcomes [7-9].

The aim of this study was to determine the magnitudes of the intrafraction prostate displacements obtained with the two immobilization systems being used in our hospital. These results would then provide guidance on the CTV-PTV margins needed during treatment planning. It is well-known that internal organ motion creates intrafraction prostate displacements [10]. Thus, intrafraction prostate displacement depends on the immobilization system and internal organ motion. The secondary aim of this study was to determine if the intrafraction prostate displacement differed between the two immobilization systems since both are commercially available and in wide clinical use.

## Materials and methods

Six consecutive patients (5 fractions per patient) with localized prostate cancer and implanted fiducial marker seeds in the prostate were selected. Patients were treated on a Novalis® (BrainLab AG, Munich, Germany) linear accelerator and were positioned using the ExacTrac6D Robotics (ETR) (BrainLab AG, Munich, Germany) system by seed matching. Three patients were immobilized with the CombiFixTM (Civco Medical Solutions, Orange City, IA) (system A) and three patients were immobilized with the BodyFIX® (Medical Intelligence GmbH, Schwabmünchen, Germany) (system B). Planar X-ray images were acquired pre-treatment once the patient was in position for treatment and post-treatment following treatment delivery. The time interval between the image acquisitions was determined by reviewing the timestamps in the record and verify system. Digitally reconstructed radiographs (DRRs) reconstructed from the computed tomography (CT) simulation and ExacTrac X-ray images were aligned either manually or automatically by matching the implanted fiducial marker seeds. The residual differences between planning DRRs and X-ray images in the vertical, longitudinal, and lateral directions were recorded for pre-treatment and post-treatment images. Differences between the pre-treatment and post-treatment positions indicate the intrafraction displacement of the prostate.

**Figure 1:**
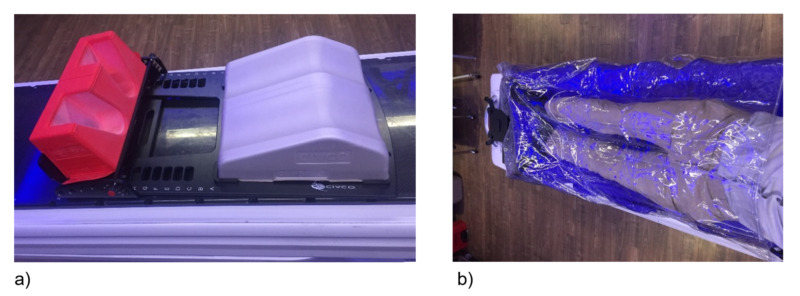
Immobilization systems a) Immobilization system A, and b) Immobilization system B

Figure 1 shows systems A and B, which have been used for prostate immobilization in our hospital. System A is a baseplate system providing enhanced positioning for the pelvic region and lower extremities. The system combines two cushions with optional elevation blocks into a fixed and indexable position. System B is a custom bag molded to the patient and a plastic covering that secures the patient to the bag using adjustable vacuum pressure. This enables accurate, precise patient positioning and immobilization.

Prostate cancer treatment delivery with the Novalis® linear accelerator takes several minutes, and during this time, the prostate may move even though the patient is in an immobilization device. For a treatment fraction, the intrafraction displacement of the prostate is equal to the difference of the post-treatment values and the pre-treatment values. The displacements were found in the lateral, longitudinal, and vertical directions. In addition, the total magnitude of the displacement was computed from these values. The mean displacements for a particular patient over the course of five fractions are found by averaging the displacements from all fractions. If there is not a systematic error, the mean displacement is expected to be zero but may be positive or negative. The average absolute displacements for a particular patient are found by averaging the absolute displacements from all fractions, which will always be positive, and similarly for the average total displacement.

The van Herk formula ($\Delta = 2.5 \Sigma + 0.7 \sigma$) was used to calculate the CTV-PTV margins (lateral, longitudinal, vertical) based on intrafraction displacements, where S is the standard deviation of the systematic error and s is the standard deviation of the random error [11]. $\Sigma$ was estimated by the standard deviation of all the patient means, $\sigma$ is the standard deviation of random error, which equals the root mean square (RMS) of the standard deviation of each patient’s mean displacements.

## Results

The average absolute displacements for patients who were immobilized with system A were 0.9 ± 0.2 mm, 1.7 ± 0.4 mm, and 1.3 ± 0.3 mm, and for patients who were immobilized with system B, they were 0.5 ± 0.2 mm, 0.6 ± 0.2 mm, and 0.8 ± 0.2 mm in the lateral, longitudinal, and vertical directions, respectively. Averaged over all patients and treatment fractions, the total three-dimensional intrafraction displacements were 2.8 ± 0.4 mm for system A and 1.3 ± 0.3 mm for system B. Table 1 shows the average absolute displacements in all directions for five fractions. The average of the total patient displacement distance from the treatment position for six patients in five fractions each is also shown in Table 1. The average time interval between imaging pre- and post-treatment varied from 10.0 to 12.8 minutes (Table 1). 

**Table 1:** Intrafraction prostate displacements Average absolute displacement in all directions and average total patient displacement distance from the treatment position for six patients over five fractions, including immobilization system used and image interval (treatment) time mean and standard deviation (SD)

	System	Image Interval = Treatment Time (min)	Average Absolute Displacement (mm)			Average Total Displacement Distance (mm)
		Mean ± SD	Lateral	Longitudinal	Vertical	
Patient #1	A	12.2 ± 0.9	0.4 ± 0.4	1.7 ± 0.8	1.6 ± 0.7	2.4 ± 1.0
Patient #2	A	10.0 ± 0.6	0.7 ± 0.3	1.6 ± 0.9	1.3 ± 0.8	2.4 ± 0.9
Patient #3	A	12.8 ± 0.4	1.5 ± 1.0	1.9 ± 0.9	1.0 ± 0.8	2.9 ± 0.7
Patient #4	B	11.6 ± 0.5	0.7 ± 0.5	0.6 ± 0.3	0.5 ± 0.1	1.1 ± 0.4
Patient #5	B	10.6 ± 0.8	0.3 ± 0.2	0.7 ± 0.6	1.0 ± 0.6	1.3 ± 0.7
Patient #6	B	12.0 ± 0.0	0.6 ± 0.4	0.5 ± 0.4	1.1 ± 0.6	1.5 ± 0.4

The averages of all the individual means that were used to calculate the systematic error are provided in Table 2. In addition, Table 2 demonstrates a higher variability of intrafraction displacements for system A, and, therefore, a lower CTV-PTV margin for system B.

**Table 2:** Intrafraction displacements of two immobilization systems Mean standard deviation (SD Σ), root mean square (RMS σ) deviations, and margin (CTV-PTV margin Δ) for intrafraction displacements of two immobilization systems

	System A				System B		
	Lateral	Longitudinal	Vertical		Lateral	Longitudinal	Vertical
Mean (mm)	-0.2	-1.3	-0.8		-0.1	0.3	-0.3
SD ($\Sigma$) (mm)	0.5	0.3	1.0		0.3	0.4	0.5
RMS ($\sigma$) (mm)	1.7	2.4	1.8		1.0	1.0	1.6
Margin ($\Delta$) (mm)	2.5	2.5	3.6		1.4	1.6	2.4

## Discussion

The intrafraction prostate motion of two immobilization systems for prostate patients was investigated. A major limitation of this report is that only six patients were accrued. Therefore, the power of statistical inference from the results is limited. However, the results are informative, as they provide insight into the performance one might expect to achieve with these systems. In order to obtain statistically significant conclusions, further investigation with more patients is necessary. Despite this limitation of having few patients, it is encouraging that the intrafraction displacements and calculated margins found here are similar, and the standard deviations of 0.3-0.5 mm for prostate displacements are smaller than those found in a large study of SBRT patients [10] using the same van Herk formulation [11]. Furthermore, the observed mean displacement in the present study is the largest in the longitudinal and vertical directions, which is also consistent with previous results [10]. These results are consistent with internal anatomical variations resulting from rectal gas, bladder filling, or coughing, creating prostate displacements that are predominantly in the longitudinal and vertical directions.

Our results indicate that there is less intrafraction displacement for patients immobilized with system B than system A. In fact, for all patients immobilized with system B, the average total displacement distance observed was less than for any patient immobilized with system A. This suggests that the observed intrafraction displacement is not entirely a result of internal organ motion. There might be an observable and significant component contributed by the immobilization system.

Although not rigorously evaluated, there were not any noticeable dosimetric differences in the treatment plans between patients immobilized with the two systems. The treatment couch is modeled in the treatment planning system and the immobilization system is included in the external contour and accounted for in the calculations.

It is important to consider a few possible confounding factors, which prohibit drawing stronger conclusions from the results. Patients were immobilized using either system A or system B, so the different systems were not compared on the same patient. The immobilization assignment was made consecutively; patients were not randomized. Each patient was observed for only five fractions. Finally, the total sample size was only three patients with each system.

The intrafraction displacements observed for patients immobilized with system B are comparable to those found during real-time tracking of the prostate [12], suggesting that for these patients, internal organ motion might be the primary cause of the displacements. If so, the performance of this immobilization system is optimal.

Several studies have investigated the necessity of immobilization devices for patient position reproducibility [13-17]. By considering average isocenter shifts, Fiorino et al. showed that an immobilization system that fixes the legs is more reproducible than an immobilization system that fixes the pelvic region [15]. In addition, patient positioning depends on the flexibility and rigidity of the immobilization system materials [18]. System A positions both the pelvis and lower legs. However, the patient is not rigidly fixed in place. System B exhibited negligible changes in shape and, in addition to positioning the pelvis and the lower legs, fixes the patient in place with pressure from a vacuum system. The observed intrafraction displacements for patients in system A show values larger than observations of intrafraction organ motion alone, while patients in system B show displacements comparable to intrafraction organ motion. This is consistent with previous immobilization studies given the physical properties of these immobilization systems [13-18].

SBRT prostate clinical trial protocols specify the CTV to PTV margins to be used in treatment planning [19-21]. While there is no consensus for standardized margin values for prostate SBRT across all trials, clinical sites participating in a given trial should have positioning and immobilization techniques that meet the specified CTV-PTV margins in the study protocol. A phase II study of prostate SBRT (35 Gy in five fractions) using volumetric modulated arc therapy and flattening free 10 MV photons, which specified CTV to PTV margins of 3-5 mm in each direction, found that the treatments were feasible and tolerated in an acute setting [19]. The PROMETHEUS study protocol, exploring the safety, efficacy, and feasibility of a two fraction SBRT boost, specifies a CTV to PTV margin of 5 mm everywhere except posterior where it is 3 mm [20]. The Novel Integration of New prostate radiation schedules with adJuvant Androgen deprivation (NINJA) clinical trial is a randomized study comparing two emerging SBRT regimens (a five fraction and a two fraction) for efficacy specifies a 3 mm uniform CTV to PTV expansion for SBRT treatments [21]. The margins found for the two systems analyzed in our study are comparable to those required in these SBRT protocols. Depending on the protocol, one or both would be acceptable for use.

## Conclusions

Patients immobilized with system A require a larger CTV-PTV margin than those with system B. Since both systems position the hips and lower legs, this suggests that rigid fixation of the patient is an important feature of an immobilization system to reduce intrafraction prostate displacement. However, the margins found for both systems are comparable to the margins used for hypofractionated prostate SBRT protocols. The margins found for system B are comparable to previously reported intrafraction internal prostate organ displacements in the literature. A real-time tracking system would be needed to reduce the margins further.
